# Update on the Impact of Omega 3 Fatty Acids on Inflammation, Insulin Resistance and Sarcopenia: A Review

**DOI:** 10.3390/ijms19010218

**Published:** 2018-01-11

**Authors:** Alex Buoite Stella, Gianluca Gortan Cappellari, Rocco Barazzoni, Michela Zanetti

**Affiliations:** Department of Medicine, Surgery, and Health Sciences, University of Trieste, Strada di Fiume 447, 34100 Trieste, Italy; alex.buoitestella@gmail.com (A.B.S.); gigici2@iol.it (G.G.C.); barazzon@units.it (R.B.)

**Keywords:** omega 3 fatty acids, sarcopenia, inflammation, aging, insulin resistance, chronic kidney disease

## Abstract

Elderly and patients affected by chronic diseases face a high risk of muscle loss and impaired physical function. Omega 3 fatty acids (FA) attenuate inflammation and age-associated muscle loss, prevent systemic insulin resistance and improve plasma lipids, potentially impacting on sarcopenia. This paper aims to review recent randomized clinical studies assessing the effects a chronic omega 3 FA supplementation on inflammatory and metabolic profile during conditions characterized by sarcopenia (aging, insulin resistance, type 2 diabetes, chronic renal failure). A comprehensive search of three online databases was performed to identify eligible trials published between 2012 and 2017. A total of 36 studies met inclusion criteria. Omega 3 FA yielded mixed results on plasma triglycerides in the elderly and no effects in renal patients. No changes in systemic insulin resistance were observed. Inflammation markers did not benefit from omega 3 FA in insulin resistant and in renal subjects while decreasing in obese and elderly. Muscle related parameters improved in elderly and in renal patients. In conclusion, in aging- and in chronic disease-associated sarcopenia omega 3 FA are promising independently of associated anabolic stimuli or of anti-inflammatory effects. The evidence for improved glucose metabolism in insulin resistant and in chronic inflammatory states is less solid.

## 1. Introduction

Declined muscle mass, functional status and metabolic demand with advancing age are prevalent in chronic disease states including the metabolic syndrome, type 2 diabetes, cancer and chronic renal failure [1,2,3,4]. These conditions are characterized by the activation of common pathways, which ultimately induce loss of muscle mass either because of blunted muscle protein synthesis or because of accelerated protein breakdown. Systemic low-grade inflammation, which characterizes disease- and age-related muscle decline induces muscle wasting by the activation of multiple pathways [5]. In addition, inflammation-induced insulin resistance may accentuate the metabolic dysfunction in skeletal muscle in the presence of preexisting type 2 diabetes mellitus (T2DM) [6]. Given that skeletal muscle accounts for up to 40% of total body mass, a significant change in its metabolic function may significantly impact systemic glucose disposal. Finally, excess oxidative damage, which is usually associated with inflammation may induce accumulation of dysfunctional proteins and DNA damage in muscle [7].

Interventions targeted at the correction of the aberrant activation of these pathways are therefore likely to preserve muscle mass and function resulting in improved systemic homeostasis, lifespan and progression of chronic age-related diseases. Clinical studies have shown that in humans specific nutrients can mechanistically interfere with the processes associated with muscle deterioration during aging and chronic diseases. In human and animal studies, omega 3 fatty acids (omega 3 FA) suppress muscle protein degradation [8], enhance the rate of muscle protein synthesis in response to anabolic stimuli (feeding or physical exercise) [9,10], quench systemic oxidative stress and inflammation [11,12], and improve insulin sensitivity and lipid profile [13]. Despite this evidence, results of clinical studies addressing the beneficial effects of omega 3 to counteract muscle mass decline are less clear-cut. 

Therefore, the aim of the current work is to review and discuss the findings from the most recent human studies to determine the effects of omega 3 FA on significant determinants of muscle mass and function, systemic inflammation, metabolic and lipid profile in age-associated chronic diseases as compared to healthy individuals.

## 2. Results

### 2.1. Metabolic and Lipid Profile in Healthy, Elderly and Chronic Renal Failure

In healthy subjects, omega 3 supplementation resulted in divergent effects on glucose and lipid reduction depending on the dose of eicosapentaenoic acid (EPA) or docosahexaenoic acid (DHA). Short term supplementation (6 weeks) of different doses omega 3 (600 mg of EPA or 1800 mg EPA or 600 mg DHA daily) was provided to 121 healthy individuals randomly allocated to a 1:1:1:1 ratio, and was compared with an olive oil treatment [14]. Main results from the pairwise placebo comparison suggest that only DHA supplementation significantly affected metabolic profile, reducing post prandial triglyceride (TG) concentration by −52.2 mg/dL (−99.3, −5.0), equal to a −20.0% (−38.7, −1.4) reduction (*p* < 0.05). DHA also increased postprandial LDL levels by 18.5 mg/dL (10.0, 27.0) (*p* < 0.01) and postprandial total cholesterol (TC) by 10.9 mg/dL (0.8, 21.0) (*p* < 0.05. EPA affected metabolic profile only when administered at high dose of 1800 mg, reducing both fasting and post prandial triglyceride-rich lipoprotein (TRL) concentration when expressed as percentage of difference from baseline, respectively by −14.6% (−27.0, −2.2) and −12.6% (−25.2, 0) (*p* < 0.05) [14]. A study in obese women receiving 360 mg EPA and 1290 mg DHA compared with placebo for 3 months, found reduced triglyceride-rich-lipoprotein levels from 1.48 ± 0.61 mmol/L to 1.22 ± 0.45 mmol/L (*p* < 0.01), and reduced insulin concentration from 16.10 ± 5.44 µIU/mL to 14.15 ± 4.37 µIU/mL (*p* < 0.05). No effect was seen on TC, high density lipoproteins (HDL), low density lipoproteins (LDL) and fasting blood glucose (FBG) [15].

In the elderly omega 3 supplementation showed contrasting results on metabolic profile. Twenty-four elderly women received 360 mg EPA and 1290 mg DHA daily for 12 weeks, or placebo. Omega 3 treatment reduced TG concentration from 1.30 ± 0.14 to 1.01 ± 0.14 mmol/L (−29%) (*p* < 0.01), while compared with no effects observed in the placebo group [16]. No effects were observed on plasma insulin or FBG. Conversely, a 1860 mg EPA and 1500 mg DHA daily supplementation for 6 months was not effective in lowering TG, HDL, LDL and FBG, in healthy elderly [17]. Fish oil administration was also evaluated with or without vitamin E (vit E) supplementation, and compared to placebo. Seventy-four women transitioning through menopause received 540 mg EPA and 360 mg DHA daily, with or without addition of 400 mg vit E, or a placebo treatment for 3 months. Results indicated a decrease in TC (−5.4% for fish oil only, −7.5% for fish oil and vit E) and LDL concentration (−8.4% for fish oil only, −7.3% for fish oil and vit E) [18].

Omega 3 supplementation in end-stage renal disease on hemodialysis has been evaluated in different studies. Fifty-two patients received for 6 months 340 mg EPA and 360 mg DHA daily, or a placebo [19]. TC, LDL, TG, serum albumin and urea were unchanged after treatment in both groups [19]. The same data were confirmed in another study, using the same supplementation protocol [20]. In contrast, a different study, assessing the effects of 1080 mg EPA and 720 mg DHA daily compared to placebo for 4 months, demonstrated decreased HDL from 48.47 ± 18.52 to 33.58 ± 7.82 mg/dL (*p* < 0.01) in the omega 3 treatment group [21]. In this group authors also found decreased TC from 167.4 ± 46.4 to 156.3 ± 57.9 mg/dL (*p* < 0.05), while post-treatment TG values were not significantly different from baseline, although lower than in placebo group by −29.11 mg/dL (*p* < 0.05). No difference was observed in FBG, LDL, homeosis model assessment insulin resistance (HOMA-IR), insulin, leptin or adiponectin [21]. No effects of higher doses of omega 3 FA (1914 mg EPA and 957 mg DHA daily supplementation compared to placebo for 12 weeks), were also observed for FBG level and HOMA-IR [22]. A small dose of omega 3, consisting in 80 mg EPA and 120 mg DHA daily for 10 weeks, was compared with a placebo condition using vit E [23]. TC was found to be significantly decreased (*p* < 0.05) in both the treatment and the placebo group, with no significant difference between groups. No effect or difference was found in TG, HDL or LDL [23]. These results have been partly confirmed also in chronic ambulatory peritoneal dialysis (CAPD) patients using a low dose of omega 3 (540 mg EPA and 360 mg DHA daily for 8 weeks), in which no effect of treatment was found on serum TG, TC, HDL and LDL concentration [24,25]. Results are summarized in Table 1.

### 2.2. Metabolic and Lipid Profile in Impaired Glucose Metabolism (IGM) and T2DM

Impaired glucose metabolism (IGM) patients with coronary artery disease received 1800 mg EPA daily compared with placebo treatment for 6 months [27]. Results indicate an increase in HDL concentration by 2.0 mg/dL (−3.0, 8.0) (*p* = 0.05), and a decrease in fasting TG by −24.0 mg/dL (−54.0, −3.0) (*p* < 0.01) after omega 3 supplementation No effect was found for TC, LDL, HOMA-IR, Hb1Ac and FBG [27]. A higher dose including also DHA, consisting in 2388 mg EPA and 1530 mg DHA, was given daily to IGM patients for 9 months, and compared to placebo [28]. Despite the higher dose, no effect was found on FBG, fasting EGB, insulin concentration, HOMA-IR during hyperinsulinaemic-euglycemic-euaminoacidaemic clamp. Nevertheless, omega 3 treatment increased total protein disposal by 9.6% and endogenous whole-body protein turnover by 10.4% under insulin-stimulated conditions (*p* < 0.01).

In T2DM, supplementation of 1000 mg EPA and 1000 mg DHA daily for 3 months was compared with a placebo group [29]. No effects were seen on insulin concentration, HbA1c, C peptide, TC, LDL, HDL, leptin and adiponectin. At baseline, omega 3 group showed significantly higher TG levels than placebo, and those levels decreased after treatment from 1.79 mmol/L (1.8, 2.41) to 1.48 mmol/L (0.91, 2.08) although without reaching significant difference from placebo group [29]. Higher doses of EPA, consisting in 1548 mg EPA and 828 mg DHA daily for 2 months, compared to placebo, decreased HbA1c from 7.90 ± 0.2% to 7.25 ± 0.17% (*p* < 0.01), while in the placebo group HbA1c increased (*p* < 0.05) [30]. In another study, a dose of 1240 mg EPA and 840 mg DHA daily for 10 weeks, decreased retinol binding protein 4 by −10.85 ± 1.62 μg/mL (*p* < 0.01) compared with placebo [31]. Omega 3 treatment (750 mg EPA and 2000 mg DHA) has been also associated with or without 15 mg pioglitazone (Pio) daily for 24 weeks [32]. HbA1c decreased significantly (*p* < 0.05) after omega 3 treatment by −7 mmol/mol (1, 13) compared with placebo and Pio groups (*p* < 0.05). In contrast, FBG increased after omega 3 treatment by 1.07 mmol/L (0.18, 2.02), but it was significantly different only from Pio (*p* < 0.05). No difference was observed in TC, HDL, LDL, NEFA, leptin, adiponectin or TG in none of the groups [32]. Diabetic patients with non-alcoholic steatohepatitis received 2160 mg EPA and 1440 mg DHA daily, or placebo, for 48 weeks [33]. Compared to baseline levels, FBG and HOMA-IR increased in omega 3 treatment (from 129.9 ± 36.5 to 150.4 ± 43.7 mg/dL and from 12.0 ± 6.8 to 16.1 ± 10.3 respectively, *p* < 0.05), with no effect in the placebo group. HbA1c also tended to increase from 6.7 ± 0.9 to 7.5 ± 2.2 (*p* = 0.059). No difference was found in TG, TC, and HDL [33]. 

Patients with T2DM or metabolic syndrome participated to a study investigating omega 3 treatment with 3580 mg EPA and 2440 mg DHA daily, botanical oil, or corn oil for 8 weeks [34]. Omega 3 supplementation increased HDL from 40.7 ± 2.8 to 43.6 ± 2.8 mg/dL (*p* < 0.01), decreased TG from 187.2 ± 22.0 to 156.8 ± 14.7 mg/dL (*p* < 0.05), increased insulin from 19.1 ± 4.5 to 24.6 ± 6.8 mg/dL (*p* < 0.05), and slightly reduced HbA1c from 7.42 ± 0.33 to 7.20 ± 0.32 (*p* = 0.05). No effect was found on TC, LDL, leptin, FBG, and HOMA-IR [34]. Treatment of metabolic syndrome patients with 1800 mg EPA and 1200 mg DHA daily was also associated with or without 10 mL of extra virgin oil for 90 days [35]. Omega 3 treatment without olive oil had no effect on TG, TC, HDL, LDL, FBG, insulin and HOMA-IR; nevertheless, in association with olive oil it reduced TC and LDL (*p* < 0.05) [35]. Omega 3 supplementation of 1800 mg EPA and 1200 mg DHA was also associated with or without 29 g of kinako or placebo for 90 days, finding decreased TG, increased TC, LDL, FBG, fasting insulin, and HOMA-IR, with no effect on HDL [36]. Results are summarized in Table 2.

### 2.3. Inflammation and Oxidative Stress in in Healthy, Elderly and Chronic Renal Failure

In healthy individuals, 6 week omega 3 daily supplementation with 600 mg EPA or 1800 mg EPA or 600 mg DHA was compared with placebo [14]. Among the inflammatory markers, only Lp-PLA2 concentration was reduced by EPA in a dose-dependent manner with a non significant decrease of −13.0 ng/mL (−28.3, 2.2) compared with placebo in the low dose treatment, and −21.4 ng/mL (−34.9, −7.8) in the high dose treatment (*p* < 0.05 vs. placebo), while no effect was seen in the DHA or placebo groups. Other inflammatory markers as hsCRP, TNF-α, IL-6, VCAM-1, ICAM-1 and fibrinogen were unaffected by the dose or type of treatment [14]. Compared to placebo, administration of 1000 mg EPA and 400 mg DHA daily for 18 weeks failed to demonstrate any effect on inflammatory status expressed as hsCRP and IL-6 concentrations in a healthy population, although hsCRP levels at baseline were significantly higher in the omega 3 group than in placebo [37]. Again, different doses of combined EPA and DHA (i.e., 300, 600, 900 and 1800 mg daily for 5 months in healthy individuals) did not result in any difference from placebo or in a dose-response effect on IL-6, TNF-α and CRP levels [38]. In another study, obese women received 360 mg EPA and 1290 mg DHA daily for 3 months [15]. Compared with placebo, reduced sVCAM-1 (from 576.86 ± 114.59 to 553.36 ± 130.25 ng/mL, *p* < 0.01), sPECAM-1 (from 71.25 ± 12.11 to 65.27 ± 8.99 ng/mL, *p* < 0.01), and hsCRP (from 3.16 ± 1.99 to 2.52 ± 1.57 mg/mL, *p* < 0.05) were observed in the treatment group. Difference was not significant between post treatment effect compared with placebo, or in IL-6 [15].

Elderly individuals with mild cognitive impairment received either 720 mg EPA and 480 mg DHA daily for 6 months, or placebo [39]. Compared to placebo, the treatment group showed reduced levels of IL-6 (−34.94 ± 46.18 pg/mL, *p* < 0.05), TNFα (−5.91 ± 9.03 fmol/mL, *p* < 0.05), and a tendency for reduced sPLA2 (−113.58 ± 249.81 ng/L, *p* = 0.052). No significant difference was observed in IL-10, COX and LOX [39]. Oxidative stress was studied in women transitioning through menopause receiving 540 mg EPA and 360 mg DHA daily, with or without addition of 400 mg vit E, or a placebo treatment for 3 months. Following EPA and DHA supplementation TBARS levels increased by 0.05 ± 0.01 μg/L, compared with both placebo (*p* < 0.01) and EPA and DHA with vit E (*p* < 0.05) [18].

In hemodialysis patients receiving for 6 months 340 mg EPA and 360 mg DHA daily, or a placebo, active treatment decreased vascular cell adhesion molecule (VCAM) from 34.1 ± 31.4 U/mL to 21.3 ± 12.9 U/mL (*p* < 0.05), while no effects where seen in the placebo group or in ICAM values [19]. In patients receiving 1080 mg EPA and 720 mg DHA daily, compared with placebo for 4 months, no effect was observed on CRP levels [21]. Similarly, no effect on hsCRP and IL-6 were observed in hemodialysis patients receiving higher dose of omega 3 (1914 mg EPA and 957 mg DHA daily), compared with placebo for 12 weeks [22]. Conversely, some effects on inflammatory status were observed in patients treated with 1080 mg EPA and 720 mg DHA daily, compared with placebo, for 4 months [40]. Authors found increased IL-10/IL-6 (by 0.64 ± 1.14 respect to baseline *p* < 0.01 and to placebo *p* < 0.05). IL-6 levels were reduced respect to baseline (by −7.53 ± 126.01, *p* < 0.05), but no difference was observed compared with placebo. No difference was observed respect to baseline or placebo in TNFα, IL-10 and CRP [41]. When omega 3 treatment with 1600 mg EPA and 300 mg DHA was associated with or without 400 IU of α-tocopherol, and compared with placebo for 12 weeks, increased plasma nitric oxide (+31.0 ± 40.0 µmol/L, *p* < 0.01), and increased total antioxidant capacity (TAC) (+57.6 ± 157.8 mmol/L, *p* < 0.01) were observed. No effect was seen on hsCRP and glutathione (GSH), while albumin and malnonyldialdehyde (MDA) levels where changed only when EPA and DHA were associated with α-tocopherol [42]. Omega 3 treatment in chronic kidney disease patients with 1840 mg EPA and 1520 mg DHA daily was also associated with or without 200 mg coenzyme Q10 (CoQ) for 8 weeks, and compared to placebo [43]. Oxidative stress was determined by F2-isoprostanes, whose values were reduced after omega 3 treatment (from 1714 to 1215 pmol/L, *p* < 0.01), with no effect of CoQ and no significant changes in placebo. No effect was seen on hsCRP concentration [43]. Moreover, no effect in inflammatory status, determined with CRP and IL-6, or oxidative stress, expressed as superoxide dismutase (SOD) and GSH, was observed in CAPD patients receiving 540 mg EPA and 360 mg DHA daily for 8 weeks/2 months compared to placebo [24,25]. Results are summarized in Table 3.

### 2.4. Inflammation and Oxidative Stress in IGM, Diabetes, and Metabolic Syndrome

Patients with IGM allocated either to treatment with 1800 mg EPA daily or to placebo for 3 months, presented in the omega 3 group reduced hsCRP concentrations compared to baseline of −0.01 mg/dL (−0.08, 0.00) (*p* < 0.01), although the same effect was observed also in the placebo group [27]. No effect on inflammatory status, expressed as IL-1β, IL-6 and hsCRP, was observed with a higher dose of omega 3 consisting in 2388 mg EPA and 1530 mg DHA daily compared to placebo for 9 months [28].

In T2DM, 1000 mg EPA and 1000 mg DHA daily, compared to placebo for 3 months, did not affect systemic inflammatory status determined by hsCRP, IL-6, and TNFα [29]. Omega 3 treatment effect was also evaluated with separated doses of 1000 mg EPA or 1000 mg DHA daily, and compared with placebo, for 12 weeks [44]. Neither EPA nor DHA significantly affected systemic inflammatory status (determined by CRP) and oxidative stress (determined by MDA) in type 1 diabetes (T1DM) patients. However, MDA increased in the placebo group, and omega 3 may help preventing MDA increase [44]. These results confirm a previous study in which patients received 900 mg EPA daily for 12 weeks and were compared to placebo, showing no effect of treatment in none of the inflammatory markers (i.e., CRP, IL-6 and TNFα) and on oxidative stress (expressed by reactive oxygen species, MDA, GSSG/GSH and SOD [45]. Omega 3 treatment with 750 mg EPA and 2000 mg DHA daily was also associated with or without 15 mg pioglitazone (Pio) for 24 weeks and compared to placebo; none of the treatments showed any effect on oxidative stress expressed by superoxide dismutase activity, TBARS and GSSG/GSH [32].

Similarly, patients with metabolic syndrome receiving 800 mg EPA and 1200 mg DHA daily with or without the addition of 10 mL extra virgin oil for 90 days showed no effect on CRP levels and oxidative stress markers when compared with placebo [35]. Results are summarized in Table 4.

### 2.5. Muscle Mass and Function, and Whole-Body Energetics in Healthy, Elderly and Chronic Renal Failure

Healthy adults received 1770 mg EPA and 390 mg DHA, 1000 IU vitamin D, or placebo daily, for 2 weeks, and the non-dominant arm was immobilised in a sling for 9 waking hours a day [46,47]. No effect of supplementations was observed in muscle thickness and upper and lower arm girths, with a trend for a smaller reduction compared to placebo [47]. Similarly, no effect of supplementations was observed in muscle elbow flexion and extension isometric and isokinetic torque, although there was a trend for an attenuated reduction compared to placebo [46]. Omega 3 supplementation with 2000 mg EPA and 1000 mg DHA was given daily to healthy active individuals, and it was compared with placebo for 12 weeks (Gerling et al., 2014). No treatment effect was observed on whole muscle, sarcolemmal, or mitochondrial fatty acid translocase (FAT/CD36), plasma membrane fatty acid binding protein; (FABPpm), fatty acid transport proteins 1 and 4 (FATP1 and FATP4), or mitochondrial electron chain and E1α subunit of pyruvate dehydrogenase (PDH) proteins, while a significant increase was observed in mitochondrial long form of uncoupling protein 3 (UCP3) by 11% (*p* < 0.05) [48].

In elderly women receiving supplementation of 360 mg EPA and 1290 mg DHA daily for 12 weeks, or placebo, omega 3 treatment resulted in increased resting metabolic rate (by 0.13 ± 0.04 kcal/min, +14%, *p* < 0.01), increased rate of fat oxidation (by 15.5 ± 5.7 mg/min, +19%, *p* < 0.01), and a decreased resting heart rate (by −3 ± 1 beats per minute, −5%, *p* < 0.05). Resting metabolic rate and resting heart rate was found to be decreased also after only 6 weeks of supplementation (*p* < 0.01) with similar magnitude to the 12 weeks effect [16]. In elderly males and females, 1880 mg EPA and 1500 mg DHA daily for 6 months, compared to placebo, increased thigh muscle volume by 3.6% (0.2, 7.0) (*p* < 0.05), handgrip strength by 2.3 kg (0.8, 3.7) (*p* < 0.01), and maximal repetition muscle strength (1-RM) by 4.0% (0.8, 3.7) (*p* < 0.05). There was also a tendency to an increase on average isokinetic muscle power after 6 months omega 3 treatment [17]. Different results were observed in elderly individuals receiving a smaller dose of omega 3 consisting in 660 mg EPA and 440 mg DHA with or without 10 mg vit E daily for 12 weeks, compared to placebo, in which no effect of any treatment was observed on muscle mass, hand grip and Timed Up and Go test (TUG) test [49].

In patients on hemodialysis receiving 1914 mg EPA and 957 mg daily, or placebo, for 12 weeks, multivariable linear regression model adjusted for baseline values showed that omega 3 supplementation significantly reduced forearm muscle protein breakdown by −57 μg/100 mL per min (−93, −27) (*p* < 0.01), this result was also significantly different from placebo (*p* < 0.01). No effect was observed on forearm muscle protein synthesis and net balance, or on whole body protein metabolism [22]. Results are summarized in Table 5.

## 3. Discussion

In the present review, we examined the effects of omega 3 fatty acids on different aspects that may affect muscle mass and function during aging and selected chronic diseases. We finally included thirty-six articles that analysed parameters related to metabolic and lipid profile, inflammation, oxidative stress and muscle mass and function. 

### 3.1. Lipid and Metabolic Profile

The effects of different doses and combinations of EPA and DHA on plasma lipids in healthy and disease populations are conflicting, with studies indicating positive, null or negative effects. In healthy subjects triglycerides were measured only in one study, finding that a small dose of 600 mg DHA was sufficient to decrease postprandial triglycerides by −20.0%, while no effect was measured in fasting triglycerides in the placebo and EPA groups [14]. These results are consistent with prior findings in the literature, supporting the conclusion that low dosage omega 3 FA supplementation (<2 g/day) hardly affect plasma triglycerides, although a linear correlation between triglyceride-lowering effect and EPA+DHA intake has been documented [50]. Moreover, in head-to-head comparison, DHA may have a stronger triglyceride-lowering effect than an equivalent dose of EPA although increasing LDL cholesterol, in line with recent evidence [51,52,53,54]. As expected, higher doses of omega 3 (EPA 360 mg and DHA 1290 mg daily) reduced by −17.6% fasting triglycerides in obese women [15]. 

In the elderly omega 3 fatty acids supplementation yielded contrasting results, with one study finding a decrease in TG concentration by −29.0% [16], while another study showing no significant difference [17] in spite of a higher dose of EPA (1860 mg compared with 360 mg in the former study) and DHA (1500 mg compared with 1290 mg), and for a longer period of treatment (6 months compared with ~3 months). The inability of omega 3 supplementation to impact plasma triglycerides in the latter study maybe attributable to very low baseline concentrations of triglycerides (0.94 vs. 1.3 mmol/L in the former study), possibly on a genetic basis, since in both studies the use of hypolipidemic drugs and high dietary intake of omega 3 were criteria of exclusion.

The response of plasma triglycerides to omega 3 fatty acid supplementation in hemodialysis patients is more consistent in the studies included in this report, showing no effect in the low dose of supplementation range (200–900 mg/day) independently from the length of supplementation (~8 weeks-6 months) [19,20,23,25,51]. Lack of effect of higher dose (1800 g) of omega 3 could be the result of inclusion of patients on statin therapy, therefore overwhelming possible beneficial effects of omega 3 [40]. With regards to total cholesterol a dose-dependent effect has been documented with a reduction of plasma concentrations with a dose of 1800 mg EPA and DHA [21] and no effect with doses <1 g/day [20,25,26]. Only one study reported decreased total cholesterol by −23.9%, with smaller dose of 200 mg EPA and DHA for 10 weeks although it should be noted that a reduction was also demonstrated in the placebo group such as not significant difference was finally observed [23]. 

In patients with metabolic disorders, data from studies using a variety of different protocols of supplementation (with doses ranging from 1800–6020 mg/day for 8 weeks–9 months) do not show unequivocal results in terms of triglyceride-lowering effects with some reporting positive results [26,33,35] while other showing no effect [28,32,33]. The reasons for these discrepant results may be attributable to different causes, including lack of exclusion of patients on concomitant therapies directly or indirectly affecting lipid profile (statins [28], metformin [32] or any hypolipidemic therapy [33]) and low numerosity of subjects included in the study groups [33,35], thereby potentially nullifying effects of omega 3 fatty acids on plasma triglycerides. A concordant HDL increase has been reported in two out of the three studies showing a triglyceride lowering effect of omega 3 treatment [27,34,36]. These studies are however in agreement in showing none or negative effect [32] on fasting plasma glucose and on systemic insulin resistance. A similar conclusion came from a recent metanalysis [55] which however suggested a potential sex-specific effect of omega 3 fatty acids on insulin sensitivity in females. For review purposes, this topic has not specifically addressed in the current work. Also omega 3 supplementation had no effect on total cholesterol in this group of patients, independently from the dose and duration of treatment [27,29,32,33,34,35,36].

### 3.2. Inflammatory Status and Oxidative Stress

In general, omega 3 treatment with doses ranging from 300 to 1800 mg for 6 weeks to 5 months showed no effects on the inflammatory markers IL-6, TNF-α, and CRP/hsCRP in healthy subjects [14,37,38]. This is somehow expected as healthy persons were characterized by low plasma concentrations of inflammatory markers to be included in those studies, and as doses up to 4 g/day of omega 3 fatty acids have not shown any impact on circulating inflammatory cytokine levels in healthy men [54]. Seminal research in the field has also well documented that in vitro only very high doses of omega 3 fatty acids (16 g/day) reduce TNFα and interleukin 1 synthesis by mononuclear cells [56]. Conversely, in obese women who received a combined omega 3 dose of 1750 mg daily and in elderly receiving a lower dose of 1200 g/day for 6 months, omega 3 treatment reduced endothelial and systemic inflammatory markers sVCAM-1, sPECAM-1, hsCRP, IL-6 and TNFα [15,38]. It should be noted that in both studies omega 3 fatty acid status was nicely documented before and after treatment, showing that, unlike in control subjects, correction of low EPA+DHA status was paralleled by decreased levels of inflammatory markers [15,39] and by similar changes in inflammatory target genes [15]. Therefore, both baseline levels of inflammatory cytokines and omega 3 fatty acid status seem to be related to a positive/neutral response to omega 3 supplementation in these conditions. The level of evidence for an anti-inflammatory effect in a systemic inflammatory disease, such as chronic renal failure with/without renal replacement therapy is less solid. No effect was seen on CRP in 5 intervention trials using omega 3 doses ranging from a minimum of 900 mg to a maximum of 3360 mg EPA and DHA for 8 weeks up to 4 months, and compared with placebo [21,22,24,40,42,43]. Interestingly, the only study assessing the effect of low dose omega 3 (1 g) but extended to 6 months of treatment reported a positive effect of supplementation on reducing VCAM levels. Therefore, given the severity of systemic inflammation in this condition and its correlations with cachexia and mortality, adequate powered studies using high doses of omega 3 fatty acids for appropriate periods of supplementation should be designed to clearly identify their effects in this population. Accordingly, oxidative stress which is mechanistically associated with inflammation in this condition [57] was unaffected by omega 3 treatment (range: 900 to 1900 mg EPA and DHA daily for 8 and 12 weeks) [24,25,42] and paradoxically menopause women receiving a low omega 3 dose consisting in 540 mg EPA and 360 mg DHA for 3 months showed increased TBARS levels by 125% [18].

Despite different omega 3 doses ranging from 900 mg EPA to ~3918 mg EPA and DHA, and different duration of treatment from 12 weeks to 9 months, no effects were seen on inflammatory status [27,28,29,35,44,45] or oxidative stress [32,35,44,45] in patients with different metabolic disorders, including IGM, T2DM and the metabolic syndrome. For some of these reports, study design might have offset potential anti-inflammatory effects of omega 3 fatty acids, including concomitant use of statins and/or ACE/Arb [27,28], low sample size/study group [29] and administered dose (<1 g) [45]. 

### 3.3. Muscle Mass and Function, and Whole-Body Energetics

Accordingly to previous studies in the literature [9], in healthy subjects high dose omega 3 supplementation did not result in any appreciable effects on muscle metabolism parameters or in muscle mass and function under basal conditions or after upper limb immobilisation in the absence of an anabolic stimuli [46,47,48]. It is thought that omega 3 fatty acids increase the rate of muscle protein synthesis in response to feeding and to resistance exercise by stimulating the mTOR-p70s6k signalling pathway [58]. Conversely, in the elderly high doses omega 3 equal/higher than 1650 mg daily resulted in increased muscle mass, function, and whole body energetics [16,17]. Those effects were not reported only in one study, maybe due to the lower omega 3 dose intake of 1100 mg daily used in the study [49]. Altogether the above cited studies in the elderly provide novel evidence suggesting that in the proper dose omega 3 fatty acids may represent a useful therapeutic strategy to overcome anabolic resistance and to treat sarcopenia also in the absence of an anabolic stimuli. 

Only one study addressing omega 3 treatment in muscle metabolism and energetics was retrieved in hemodialysis patients with systemic inflammation (CRP ≥ 5 mg/L) who received high dose omega 3 (2.9 g/day). While omega 3 FA did not affect forearm net protein balance or synthesis, and whole body protein synthesis or breakdown, a significant reduction of forearm muscle protein breakdown was observed [22]. Interestingly, the improvement in muscle protein degradation was not accompanied by significant changes in markers of systemic inflammation, which is thought to be responsible for increased protein breakdown in these conditions [59]. This is not surprising though, as local changes in inflammatory markers could not be entirely reflected by their circulating levels and longer period of supplementation maybe required to change systemic inflammatory status.

### 3.4. Limitations and Future Perspectives

This paper presents findings generated only from the studies published in the last 5 years. While the novelty of this review lies on presenting the most recent updates on omega 3 fatty acids supplementation on lipid profile, inflammatory status and muscle metabolism, the choice of focusing on this period might have excluded previous results possibly disagreeing with our conclusions. Additionally, authors are aware that including studies in which omega 3 supplementation was different in terms of daily dose of EPA and DHA administered, duration of treatment, concomitant therapy and lack of information on basal omega 3 status may contribute to the variability of results; these issues when comparing omega 3 clinical trials have been addressed by a recent publication [60]. Nevertheless, as a narrative review, this paper aims to present an extensive overview, although with specific design criteria, of the most recent findings in RCT trials using omega 3 supplementation and its effects on health-related parameters in physiological, paraphyisiological, and systemic inflammatory conditions. Future systematic analysis are suggested to precisely define the role of treatment components, as dose and duration, on the outlined parameters in such populations.

## 4. Methods

**Figure 1:**
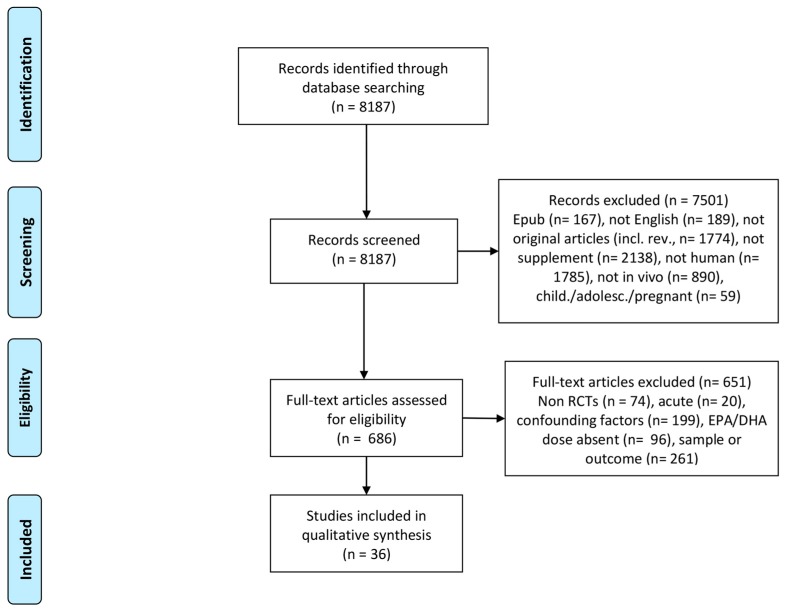
PRISMA 2009 Flow Diagram for search of literature and assessment of eligibility for studies included in the review. A total of 8187 records were identified and screened, among which 36 papers were included in qualitative synthesis and presented.

An extensive literature search was performed up to July 2017 on PubMed, Google Scholar and EMBASE, with the key words “n3 PUFAs” OR “Omega 3”, in order to obtain data about the use of omega 3 in different diseases and protocols. The searches have been re-run just before the final analysis and further studies retrieved for inclusion. A total of 25,402 studies were found. To report only the most recent results, search was then restricted to last 5 years, with a total of 8187 papers. Automatic filters were not used in literature search, except for “last 5 years”. This time frame was selected to provide information arising only from the most recent published papers, in order not to overlap results with other available and presented in previous reviews. Two independent researchers screened the papers and assessed eligibility for analysis. In case of contrasting selection, a third independent researcher with experience on omega 3 FA and external to the review was invited to have final decision. In this review, studies conducted in humans and in vivo were considered, and only when omega 3 was given as a supplement. Hence, records were excluded based on different criteria, as: epub ahead of print (167), not in english (189), errata, comments, responses, or editorials (232), omega 3 not as supplement (2138), not in humans (1785), not in vivo (890), reviews, meta-analysis and position stands (1541). Studies that were not excluded in the first screening and were assessed for eligibility were then controlled for design, treatment, population (physiological or disease condition) and outcome. Only blinded randomized clinical trials (RCTs) were considered in this review, excluding (74) papers. To consider results from a similar population and on comparable of doses of supplements, children, adolescents and pregnant women were excluded (559). To obtain data about chronic supplementation with omega 3, it was decided a cut off of minimum 2 week of duration, and outcome should not refer to acute stressors (20). In addition, all the studies in which omega 3 supplement was not the only intervention (e.g., diet, physical activity or other antioxidants) were excluded from collection (199). Only medications necessary for the treatment of the disease, or that were assigned before the study, were permitted. Omega 3 FA are a group of polyunsaturated fatty acids defined by a double bond at the third carbon from the methyl end of the carbon chain. This review focuses on eicosapentaenoic acid (20:5, EPA) and docosahexaenoic acid (22:6, DHA), two of the most common omega 3 FA found in fish oil [61]. Consequently, studies were excluded if other omega 3 fatty acids were used (72), or if the precise dose of EPA and DHA was not declared (i.e., all the studies reporting only generic omega 3 FA dose) (24). Only oral administration was considered for this survey. Since the variety of pathological conditions observed, and the extent of knowledge of some of those diseases, this review focuses on physiological (healthy individuals) and para-physiological conditions (ageing), on a systematic inflammatory disease such as renal disease, and on some metabolic disorders (IGM and T2DM). Lastly, outcome was considered for markers of metabolic profile, inflammation, and muscle mass and function, and whole-body energetics. Papers selection flow chart is shown in Figure 1.

This paper presents studies as a narrative review, and results for included studies are presented as percentage of difference from basal values if significant intra-sample (time effect) and/or inter-sample difference (treatment effect), with a significance level of *p* < 0.05.

## 5. Conclusions

There is a rationale for the use of omega 3 fatty acids in conditions characterized by muscle loss, such as aging-associated sarcopenia and CKD-associated muscle wasting. In these settings, the effect of omega 3 to increase muscle mass and to prevent muscle catabolism seems to be independent of anabolic stimuli or of anti-inflammatory effects. The use of omega 3 to counteract inflammation, oxidative stress and to improve systemic insulin resistance in healthy, insulin resistant and elderly subjects is not likely to provide any benefit.

## Figures and Tables

**Table 1:** Metabolic and lipid profile in healthy, ageing and chronic renal failure.

Study	Sample (*n*)	Protocol	Key Findings
Asztalos et al., 2016 [14]	Healthy (121)	600 EPA mg vs. 1800 mg EPA vs. 600 mg DHA daily vs. placebo, 6 weeks	High dose EPA decreased TRL fasted (−14.6%) and TRL post prandial (−12.6%) DHA decreased postprandial TG (−20.0%), and increased fasting and postprandial LDL (18.4%) No effect of low dose EPA
Polus et al., 2016 [15]	Obese women (59)	360 mg EPA and 1290 mg DHA daily vs. placebo, 3 months	Decreased fasting TG (−17.6%) and insulin (−12.1%) No effect on NEFA, TC, HDL, LDL, FBG
Logan & Spriet, 2015 [16]	Elderly women (24)	360 mg EPA + 1290 mg DHA daily vs. placebo, 12 weeks	Decreased TG (−29%) No effect on insulin or FBG
Smith et al., 2015 [17]	Elderly (60)	1860 mg EPA + 1500 mg DHA daily vs. placebo, 6 months	No effect on TG, HDL, LDL, FBG
Alves Luzia et al., 2015 [18]	Menopause women (74)	540 mg EPA + 360 mg DHA + 400 vit E (or placebo) daily vs. placebo, 3 months	Decreased TC in omega 3 group (−5.4%), omega 3 + vit E (−7.5%) and control (−1.0%). Decreased LDL in omega 3 (−8.4%) and omega 3 + vit E (−7.3%), increased LDL in control (8.3%)
Moeinzadeh et al., 2016 [19]	Hemodialysis (52)	540 mg EPA + 360 mg DHA daily vs. placebo, 6 months	No effect on serum albumin, LDL, TC, and TG
Kajbaf et al., 2016 [20]	Hemodialysis (54)	540 mg EPA + 360 mg DHA daily vs. placebo, 6 months	Increased HDL (27.5%) and increased urea reduction ratio (1.54%) No effect on serum albumin, LDL, TC, and TG
Gharekhani et al., 2016 [21]	Hemodialysis (54)	1080 mg EPA + 720 mg DHA daily vs. placebo, 4 months	Decreased serum TC (−5.37%) and HDL (−30.9%). TG reduced only compared to placebo No effect on insulin, leptin, adiponectin, FPG, LDL, HOMA-IR
Deger et al., 2016 [22]	Hemodialysis (20)	1914 mg EPA + 957 mg DHA daily vs. placebo, 12 weeks	No effect on FBG and HOMA-IR
Omrani et al., 2015 [23]	Hemodialysis (60)	80 mg EPA + 120 mg DHA daily vs. placebo, 10 weeks	Reduced TC in both experimental (−23.9%) and control (−7.7%) group No effect on HDL, LDL, and TG
Naini et al., 2015 [26]	CAPD (90)	540 mg EPA + 360 mg DHA daily vs. placebo, 8 weeks	No effect on serum TG, TC, HDL, and LDL
Taheri et al., 2014 [25]	CAPD (90)	540 mg EPA + 360 mg DHA daily vs. placebo, 8 weeks	No effect on lipid profile

TRL: triglyceride-rich lipoproteins; TG: triglycerides; LDL: low density lipoprotein; HDL: high density lipoprotein; NEFA: non esterified fatty acids; FBG: fasting blood glucose; TC: total cholesterol; HOMA-IR: homeostasis model assessment insulin resistance; CAPD: chronic ambulatory peritoneal dialysis.

**Table 2:** Metabolic profile in diabetes and metabolic syndrome.

Study	Sample (*n*)	Protocol	Key Findings
Sawada et al., 2016 [27]	IGM (107)	1800 mg EPA daily vs. placebo, 6 months	Increased HDL (5.1%) and reduced fasting TG (−25.3%) No effect on HbA1c and FBG
Clark et al., 2016 [28]	IGM (36)	2388 mg EPA + 1530 mg DHA daily vs. placebo, 9 months	Increased total protein disposal (9.6%) and endogenous whole-body protein turnover (10.4%) under insulin-stimulated conditions No effect on FBG, insulin, HOMA-IR. No effect on total glucose disposal during hyperinsulinaemic-euglycaemic-euaminoacidaemic clamp
Poreba et al., 2017 [29]	T2DM (74)	1000 mg EPA + 1000 mg DHA daily vs. placebo, 3 months	No effect on insulin, HbA1c, adiponectin, leptin, and lipid levels
Toorang et al., 2016 [30]	T2DM (90)	1548 mg EPA + 828 mg DHA daily vs. placebo, 2 months	Decreased HbA1c (−8.2%)
Farahbakhsh-Farsi et al., 2016 [31]	T2DM (45)	310 mg EPA + 210 mg DHA daily vs. placebo, 10 weeks	Reduced retinol-binding protein 4 (−42.5%)
Veleba et al., 2015 [32]	T2DM (60)	750 mg EPA and 2000 mg DHA + 15 mg Pio (or PLACEBO) daily vs. 15 mg Pio daily vs. PLACEBO, 24 weeks	Increased HbA1c (14.7%) and FBG (17.0%) No effect on TG, TC, HDL, LDL, NEFA, Leptin, Adiponectin
Dasarthy et al., 2015 [33]	T2DM with NASH (37)	2160 mg EPA + 1440 mg DHA daily vs. placebo,48 weeks	Increased FBG (15.8%), HOMA (34.2%), and HbA1c (6.4%) No effect on TG, HDL and TC
Lee et al., 2014 [34]	MetS (59)	3580 mg EPA + 2440 mg DHA daily vs. placebo, 8 weeks	Reduced TG (−16.0%) and HbA1c (−3.0%), increased insulin (29.8%) and HDL (7.1%)
Venturini et al., 2015 [35]	MetS (102)	1800 mg EPA + 1200 mg DHA + 10 mL extra virgin oil (or PLACEBO) daily vs. 10 mL extra virgin oil daily vs. placebo, 90 days	No effect on TG, TC, HDL, LDL, FBG, insulin, HOMA-IR
Simao et al., 2014 [36]	MetS (65)	1800 mg EPA + 1200 mg DHA + 29 g kinako (or PLACEBO) daily vs. 29 g kinako daily vs. placebo, 90 days	Decreased TG (−20.0%), increased TC (7.7%), LDL (18.6%), FBG (10.5%), fasting insulin (18.3%), and HOMA-IR (13.4%) No effect on HDL

TG: triglycerides; HDL: high density lipoprotein; FBG: fasting blood glucose; TC: total cholesterol; HOMA-IR: homeostasis model assessment insulin resistance; IGM: impaired glucose metabolism; T2DM: type 2 diabetes mellitus; NASH: non-alcoholic steathohepatytis; MetS: metabolic syndrome; LDL: low density lipoproteins; NEFA: non esterified fatty acids; HDL: high density lipoproteins.

**Table 3:** Inflammation and oxidative stress in healthy, ageing and chronic renal failure.

Author (Year)	Sample (n)	Treatment	Key Findings
Asztalos et al., 2016 [14]	Healthy (121)	600 mg EPA vs. 1800 mg EPA vs. 600 mg DHA daily vs. placebo, 6 weeks	High dose EPA reduced Lp-PLA2 (−14.1%) No effect of low dose EPA or DHA
Muldoon et al., 2016 [37]	Healthy (261)	1000 mg EPA + 400 mg DHA daily vs. placebo, 18 weeks	No effect on serum CRP and IL-6
Flock et al., 2014 [38]	Healthy (125)	300 mg EPA + DHA vs. 600 mg EPA + DHA vs. 900 mg EPA + DHA vs. 1800 mg EPA + DHA daily vs. placebo, 5 months	No dose-response effect on IL-6, TNF-α and CRP
Polus et al., 2016 [15]	Obese women (59)	360 mg EPA and 1290 mg DHA daily vs. placebo, 3 months	Decreased sVCAM-1 (−4.1%), sPECAM-1 (−8.4%) and hsCRP (−20.3%) No effect on IL-6
Bo et al., 2017 [39]	Elderly with mild cognitive impairment (86)	720 mg EPA + 480 mg DHA daily vs. placebo, 6 months	Decreased IL-6 (−29.0%), TNF-α (−31.1%), and sPLA2 activity (−11.3%) No effect on IL-10, COX and LOX
Alves Luzia et al., 2015 [18]	Menopause women (74)	540 mg EPA + 360 mg DHA + 400 mg vit E (or placebo) daily vs. placebo, 3 months	Increased TBARS (125%)
Moeinzadeh et al., 2016 [19]	Hemodialysis (52)	540 mg EPA + 360 mg DHA daily vs. placebo, 6 months	Decreased VCAM (−37.5%)
Gharekhani et al., 2016 [21]	Hemodialysis (54)	1080 mg EPA + 720 mg DHA daily vs. placebo, 4 months	No effect on CRP
Deger et al., 2016 [22]	Hemodialysis (20)	1914 mg EPA + 957 mg DHA daily vs. placebo, 12 months	No effect on serum hsCRP and IL-6
Gharekhani et al., 2014 [41]	Hemodialysis (54)	1080 mg EPA + 720 mg DHA daily vs. placebo, 4 months	Increased IL10 to IL-6 ratio (48.5%), reduced IL-6 (−5.2%)
Asemi et al., 2016 [42]	Hemodialysis (120)	1600 mg EPA + 300 mg DHA + 400 IU Alphatocopherol (or placebo) daily vs. placebo, 12 weeks	Increased NO (59.3%) and TAC (4.77%) No effect on albumin, hsCRP, GSH, and MDA
Barden et al., 2016 [43]	CKD (85)	1840 mg EPA + 1520 mg DHA + 200 mg CoQ (or PLACEBO) daily vs. placebo, 8 weeks	Reduced F2-isoprostanes (−29.1%)
Naini et al., 2015 [24]	CAPD (40)	540 mg EPA + 360 mg DHA daily vs. placebo, 2 months	No effect on CRP and IL-6
Taheri et al., 2014 [25]	CAPD (90)	540 mg EPA + 360 mg DHA daily vs. placebo, 8 weeks	No effect on SOD and GSH

Lp-PLA2: lipoprotein-associated phospholipase A2; IL-6: interleukin 6; IL-10: interleukin 10; TNF-α: tumor necrosis factor α; hsCRP: high sensitive C reactive protein; VCAM: vascular cell adhesion molecule; PECAM: platelet and endothelial cell adhesion molecule; COX: cyclooxygenase; LOX: lypoooxigenase; TBARS: thiobarbituric acid substances; NO: nitric oxide; TAC: total antioxidant capacity; SOD: superoxide dismutase; GSH: glutathione peroxidase; MDA: malonyldialdehyde; CKD: chronic kidney disease; CAPD: chronic ambulatory peritoneal disysis.

**Table 4:** Inflammation and oxidative stress in in diabetes and metabolic syndrome.

Author (Year)	Sample (*n*)	Treatment	Key Findings
Sawada et al., 2016 [27]	IGM (2016)	1800 mg EPA daily vs. placebo, 6 months	Reduced CRP (−10%), similar effects in placebo
Clark et al., 2016 [28]	IGM (36)	2388 mg EPA + 1530 mg DHA daily vs. placebo, 9 months	No effect in IL-1B, IL-6, hsCRP, sICAM and VCAM
Poreba et al., 2017 [29]	T2DM (74)	1000 mg EPA + 1000 mg DHA daily vs. placebo, 3 months	No effect on markers of systemic inflammation
Azizi-Soleiman et al., 2013 [44]	T2DM (60)	1000 mg EPA vs. 1000 mg DHA daily vs. placebo, 12 weeks	No effect on serum CRP and MDA
Mocking et al., 2012 [45]	T2DM (24)	900 mg EPA daily vs. placebo, 12 weeks	No effect on oxidative stress and inflammatory parameters
Veleba et al., 2015 [32]	T2DM (60)	2800 mg EPA + DHA + 15 mg Pio (or placebo) vs. 15 mg Pio daily vs. placebo, 24 weeks	No effect on SOD, TBARS, GSSG/GSH
Venturini et al., 2015 [35]	MetS (102)	1800 mg EPA + 1200 mg DHA + 10 mL extra virgin oil (or placebo) vs. 10 mL extra viring oil daily vs. placebo, 90 days	No effect on CRP and oxidative stress parameters

CRP: C reactive protein; IL-6: interleukin 6; ICAM: intercellular adhesion molecule; VCAM: vascular cell adhesion molecule; MDA: malonyldialdehyde; T2DM: type 2 diabetes mellitus; MetS: metabolic syndrome; Pio: pioglitazone; MDA: malonyldiahaldehyde; SOD: superoxide dismutase; TBARS: thiobarbituric acid substances; GSSG/GSH; oxidized/reduced glutathione.

**Table 5:** Muscle mass and function, and whole-body energetics.

Author (Year)	Sample (*n*)	Treatment	Key findings
Bostock et al., 2017a and 2017b [46,47]	Healthy (24)	1770 mg EPA + 390 mg DHA vs. 1000 IU vit D daily vs. placebo, 2 weeks	No effect on declines in muscle thickness and torque associated with immobilisation
Gerling et al., 2014 [48]	Healthy (30)	2000 mg EPA + 1000 mg DHA daily vs. placebo, 12 weeks	Increased long form of UCP3 (11%) No effect on whole muscle, sarcolemmal, or mitochondrial FAT/CD36, FABPpm, FATP1 and FATP4, or mitochondrial electron chain and PDH proteins
Logan and Spriet, 2015 [16]	Elderly women (24)	360 mg EPA + 1290 mg DHA daily vs. placebo, 12 weeks	Increased resting metabolic rate (14%), energy expenditure during exercise (10%), rate of fat oxidation during rest (19%) and during exercise (27%), increased lean body mass (4%) and functional capacity (7%)
Smith et al., 2015 [17]	Elderly (60)	1860 mg EPA + 1500 mg DHA daily vs. placebo, 6 months	Increased thigh muscle volume (3.6%), handgrip strength (6.6%), and 1-RM muscle strength (4.0%)
Lrzyminska-Siemaszko et al., 2015 [49]	Elderly (53)	660 mg EPA + 440 mg DHA + 10 mg vit E vs. placebo + 10 mg vit E, 12 weeks	No effect on muscle mass, hand grip, and TUG
Deger et al., 2016 [22]	Hemodialysis (20)	1914 mg EPA + 957 mg DHA daily vs. placebo, 12 weeks	Decreased forearm muscle protein breakdown (−42.5%) No effect on forearm muscle net protein balance or synthesis, and whole-body protein synthesis or breakdown

UCP3: uncoupling protein 3; FAT/CD36: fatty acid translocase; FABPpm: plasma membrane fatty acid binding protein; FATP1 and FATP4: fatty acid transport proteins 1 and 4; PDH: E1α subunit of PDH; 1-RM: 1 maximal repetition strength; TUG: Timed Up and Go test.

## References

[1] Sinclair A.J., Abdelhafiz A.H., Rodriguez-Manas L. (2017). Frailty and sarcopenia—Newly emerging and high impact complications of diabetes. J. Diabetes Complicat..

[2] Chow L.S., Nair K.S. (2005). Sarcopenia of male aging. Endocrinol. Metab. Clin. N. Am..

[3] Johns N., Stephens N.A., Fearon K.C.H. (2013). Muscle wasting in cancer. Int. J. Biochem. Cell Biol..

[4] Cohen S., Nathan J.A., Goldberg A.L. (2015). Muscle wasting in disease: Molecular mechanisms and promising therapies. Nat. Rev. Drug Discov..

[5] Jo E., Lee S.-R., Park B.-S., Kim J.-S. (2012). Potential mechanisms underlying the role of chronic inflammation in age-related muscle wasting. Aging Clin. Exp. Res..

[6] Wu H., Ballantyne C.M. (2017). Skeletal muscle inflammation and insulin resistance in obesity. J. Clin. Investig..

[7] Jackson M.J. (2016). Reactive oxygen species in sarcopenia: Should we focus on excess oxidative damage or defective redox signalling?. Mol. Asp. Med..

[8] Oh S.-L., Lee S.-R., Kim J.-S. (2017). Effects of conjugated linoleic acid/n-3 and resistance training on muscle quality and expression of atrophy-related ubiquitin ligases in middle-aged mice with high-fat dietinduced obesity. J. Exerc. Nutr. Biochem..

[9] Smith G.I., Atherton P., Reeds D.N., Mohammed B.S., Rankin D., Rennie M.J., Mittendorfer B. (2011). Omega-3 polyunsaturated fatty acids augment the muscle protein anabolic response to hyperinsulinaemia-hyperaminoacidaemia in healthy young and middle-aged men and women. Clin. Sci..

[10] Di Girolamo F.G., Situlin R., Mazzucco S., Valentini R., Toigo G., Biolo G. (2014). Omega-3 fatty acids and protein metabolism: Enhancement of anabolic interventions for sarcopenia. Curr. Opin. Clin. Nutr. Metab. Care.

[11] Farias J.G., Molina V.M., Carrasco R.A., Zepeda A.B., Figueroa E., Letelier P., Castillo R.L. (2017). Antioxidant Therapeutic Strategies for Cardiovascular Conditions Associated with Oxidative Stress. Nutrients.

[12] Calder P.C. (2017). Omega-3 fatty acids and inflammatory processes: From molecules to man. Biochem. Soc. Trans..

[13] Lucero D., Olano C., Bursztyn M., Morales C., Stranges A., Friedman S., Macri E.V., Schreier L., Zago V. (2017). Supplementation with n-3, n-6, n-9 fatty acids in an insulin-resistance animal model: Does it improve VLDL quality?. Food Funct..

[14] Asztalos I.B., Gleason J.A., Sever S., Gedik R., Asztalos B.F., Horvath K.V., Dansinger M.L., Lamon-Fava S., Schaefer E.J. (2016). Effects of eicosapentaenoic acid and docosahexaenoic acid on cardiovascular disease risk factors: A randomized clinical trial. Metabolism.

[15] Polus A., Zapala B., Razny U., Gielicz A., Kiec-Wilk B., Malczewska-Malec M., Sanak M., Childs C.E., Calder P.C., Dembinska-Kiec A. (2016). Omega-3 fatty acid supplementation influences the whole blood transcriptome in women with obesity, associated with pro-resolving lipid mediator production. Biochim. Biophys. Acta.

[16] Logan S.L., Spriet L.L. (2015). Omega-3 Fatty Acid Supplementation for 12 Weeks Increases Resting and Exercise Metabolic Rate in Healthy Community-Dwelling Older Females. PLoS ONE.

[17] Smith G.I., Julliand S., Reeds D.N., Sinacore D.R., Klein S., Mittendorfer B. (2015). Fish oil-derived n-3 PUFA therapy increases muscle mass and function in healthy older adults. Am. J. Clin. Nutr..

[18] Alves Luzia L., Mendes Aldrighi J., Teixeira Damasceno N.R., Rodrigues Sampaio G., Aparecida Manolio Soares R., Tande Silva I., De Queiroz Mello A.P., Ferreira Carioca A.A., Ferraz da Silva Torres E.A. (2015). Fish oil and vitamin e change lipid profiles and anti-LDL-antibodies in two different ethnic groups of women transitioning through menopause. Nutr. Hosp..

[19] Moeinzadeh F., Shahidi S., Mortazavi M., Dolatkhah S., Kajbaf M., Haghjooy Javanmard S., Moghtaderi A. (2016). Effects of Omega-3 Fatty Acid Supplementation on Serum Biomarkers, Inflammatory Agents, and Quality of Life of Patients on Hemodialysis. Iran. J. Kidney Dis..

[20] Kajbaf M.H., Khorvash F., Mortazavi M., Shahidi S., Moeinzadeh F., Farajzadegan Z., Tirani S.A. (2016). Does Omega-3 supplementation decrease carotid intima-media thickening in hemodialysis patients?. J. Res. Pharm. Pract..

[21] Gharekhani A., Dashti-Khavidaki S., Lessan-Pezeshki M., Khatami M.-R. (2016). Potential Effects of Omega-3 Fatty Acids on Insulin Resistance and Lipid Profile in Maintenance Hemodialysis Patients: A Randomized Placebo-Controlled Trial. Iran. J. Kidney Dis..

[22] Deger S.M., Hung A.M., Ellis C.D., Booker C., Bian A., Chen G., Abumrad N.N., Ikizler T.A. (2016). High Dose Omega-3 Fatty Acid Administration and Skeletal Muscle Protein Turnover in Maintenance Hemodialysis Patients. Clin. J. Am. Soc. Nephrol..

[23] Omrani H.R., Pasdar Y., Raisi D., Najafi F., Esfandiari A. (2015). The effect of omega-3 on serum lipid profile in hemodialysis patients. J. Ren. Inj. Prev..

[24] Naini A.E., Asiabi R.E.K., Keivandarian N., Moeinzadeh F. (2015). Effect of omega-3 supplementation on inflammatory parameters in patients on chronic ambulatory peritoneal dialysis. Adv. Biomed. Res..

[25] Taheri S., Keyvandarian N., Moeinzadeh F., Mortazavi M., Naini A.E. (2014). The effect of omega-3 fatty acid supplementation on oxidative stress in continuous ambulatory peritoneal dialysis patients. Adv. Biomed. Res..

[26] Naini A.E., Keyvandarian N., Mortazavi M., Taheri S., Hosseini S.M. (2015). Effect of Omega-3 fatty acids on blood pressure and serum lipids in continuous ambulatory peritoneal dialysis patients. J. Res. Pharm. Pract..

[27] Sawada T., Tsubata H., Hashimoto N., Takabe M., Miyata T., Aoki K., Yamashita S., Oishi S., Osue T., Yokoi K. (2016). Effects of 6-month eicosapentaenoic acid treatment on postprandial hyperglycemia, hyperlipidemia, insulin secretion ability, and concomitant endothelial dysfunction among newly-diagnosed impaired glucose metabolism patients with coronary artery disease. A. Cardiovasc. Diabetol..

[28] Clark L.F., Thivierge M.C., Kidd C.A., McGeoch S.C., Abraham P., Pearson D.W.M., Horgan G.W., Holtrop G., Thies F., Lobley G.E. (2016). Fish oil supplemented for 9 months does not improve glycaemic control or insulin sensitivity in subjects with impaired glucose regulation: A parallel randomised controlled trial. Br. J. Nutr..

[29] Poreba M., Mostowik M., Siniarski A., Golebiowska-Wiatrak R., Malinowski K.P., Haberka M., Konduracka E., Nessler J., Undas A., Gajos G. (2017). Treatment with high-dose n-3 PUFAs has no effect on platelet function, coagulation, metabolic status or inflammation in patients with atherosclerosis and type 2 diabetes. Cardiovasc. Diabetol..

[30] Toorang F., Djazayery A., Djalali M. (2016). Effects of Omega-3 Fatty Acids Supplement on Antioxidant Enzymes Activity in Type 2 Diabetic Patients. Iran. J. Public Health.

[31] Farahbakhsh-Farsi P., Djazayery A., Eshraghian M.R., Koohdani F., Zarei M., Javanbakht M.H., Derakhshanian H., Djalali M. (2016). Effect of Omega-3 Supplementation on Lipocalin 2 and Retinol-Binding Protein 4 in Type 2 Diabetic Patients. Iran. J. Public Health.

[32] Veleba J., Kopecky J.J., Janovska P., Kuda O., Horakova O., Malinska H., Kazdova L., Oliyarnyk O., Skop V., Trnovska J. (2015). Combined intervention with pioglitazone and n-3 fatty acids in metformin-treated type 2 diabetic patients: Improvement of lipid metabolism. Nutr. Metab..

[33] Dasarathy S., Dasarathy J., Khiyami A., Yerian L., Hawkins C., Sargent R., McCullough A.J. (2015). Double-blind randomized placebo-controlled clinical trial of omega 3 fatty acids for the treatment of diabetic patients with nonalcoholic steatohepatitis. J. Clin. Gastroenterol..

[34] Lee T.C., Ivester P., Hester A.G., Sergeant S., Case L.D., Morgan T., Kouba E.O., Chilton F.H. (2014). The impact of polyunsaturated fatty acid-based dietary supplements on disease biomarkers in a metabolic syndrome/diabetes population. Lipids Health Dis..

[35] Venturini D., Simao A.N.C., Urbano M.R., Dichi I. (2015). Effects of extra virgin olive oil and fish oil on lipid profile and oxidative stress in patients with metabolic syndrome. Nutrition.

[36] Simao A.N.C., Lozovoy M.A.B., Dichi I. (2014). Effect of soy product kinako and fish oil on serum lipids and glucose metabolism in women with metabolic syndrome. Nutrition.

[37] Muldoon M.F., Laderian B., Kuan D.C.H., Sereika S.M., Marsland A.L., Manuck S.B. (2016). Fish oil supplementation does not lower C-reactive protein or interleukin-6 levels in healthy adults. J. Intern. Med..

[38] Flock M.R., Skulas-Ray A.C., Harris W.S., Gaugler T.L., Fleming J.A., Kris-Etherton P.M. (2014). Effects of supplemental long-chain omega-3 fatty acids and erythrocyte membrane fatty acid content on circulating inflammatory markers in a randomized controlled trial of healthy adults. Prostaglandins Leukot. Essent. Fatty Acids.

[39] Bo Y., Zhang X., Wang Y., You J., Cui H., Zhu Y., Pang W., Liu W., Jiang Y., Lu Q. (2017). The n-3 Polyunsaturated Fatty Acids Supplementation Improved the Cognitive Function in the Chinese Elderly with Mild Cognitive Impairment: A Double-Blind Randomized Controlled Trial. Nutrients.

[40] Gharekhani A., Khatami M.-R., Dashti-Khavidaki S., Razeghi E., Noorbala A.-A., Hashemi-Nazari S.-S., Mansournia M.-A. (2014). The effect of omega-3 fatty acids on depressive symptoms and inflammatory markers in maintenance hemodialysis patients: A randomized, placebo-controlled clinical trial. Eur. J. Clin. Pharmacol..

[41] Gharekhani A., Khatami M.-R., Dashti-Khavidaki S., Razeghi E., Abdollahi A., Hashemi-Nazari S.-S., Mansournia M.-A. (2014). Potential effects of omega-3 fatty acids on anemia and inflammatory markers in maintenance hemodialysis patients. DARU J. Pharm. Sci..

[42] Asemi Z., Soleimani A., Shakeri H., Mazroii N., Esmaillzadeh A. (2016). Effects of omega-3 fatty acid plus alpha-tocopherol supplementation on malnutrition-inflammation score, biomarkers of inflammation and oxidative stress in chronic hemodialysis patients. Int. Urol. Nephrol..

[43] Barden A., O’Callaghan N., Burke V., Mas E., Beilin L.J., Fenech M., Irish A.B., Watts G.F., Puddey I.B., Huang R.-C. (2016). n-3 Fatty Acid Supplementation and Leukocyte Telomere Length in Patients with Chronic Kidney Disease. Nutrients.

[44] Azizi-Soleiman F., Jazayeri S., Eghtesadi S., Rajab A., Heidari I., Vafa M.R., Gohari M.R. (2013). Effects of pure eicosapentaenoic and docosahexaenoic acids on oxidative stress, inflammation and body fat mass in patients with type 2 diabetes. Int. J. Prev. Med..

[45] Mocking R.J.T., Assies J., Bot M., Jansen E.H.J.M., Schene A.H., Pouwer F. (2012). Biological effects of add-on eicosapentaenoic acid supplementation in diabetes mellitus and co-morbid depression: A randomized controlled trial. PLoS ONE.

[46] Bostock E.L., Morse C.I., Winwood K., McEwan I.M., Onambele G.L. (2017). Omega-3 Fatty Acids and Vitamin D in Immobilisation: Part B- Modulation of Muscle Functional, Vascular and Activation Profiles. J. Nutr. Health Aging.

[47] Bostock E.L., Morse C.I., Winwood K., McEwan I.M., Onambélé-Pearson G.L. (2017). Omega-3 fatty acids and vitamin D in immobilisation: Part A—Modulation of appendicular mass content, composition and structure. J. Nutr. Health Aging.

[48] Gerling C.J., Whitfield J., Mukai K., Spriet L.L. (2014). Variable effects of 12 weeks of omega-3 supplementation on resting skeletal muscle metabolism. Appl. Physiol. Nutr. Metab..

[49] Krzyminska-Siemaszko R., Czepulis N., Lewandowicz M., Zasadzka E., Suwalska A., Witowski J., Wieczorowska-Tobis K. (2015). The Effect of a 12-Week Omega-3 Supplementation on Body Composition, Muscle Strength and Physical Performance in Elderly Individuals with Decreased Muscle Mass. Int. J. Environ. Res. Public Health.

[50] Mozaffarian D., Rimm E.B. (2006). Fish intake, contaminants, and human health: Evaluating the risks and the benefits. JAMA.

[51] Sharp R.P., Gales B.J., Sirajuddin R. (2017). Comparing the Impact of Prescription Omega-3 Fatty Acid Products on Low-Density Lipoprotein Cholesterol. Am. J. Cardiovasc. Drugs.

[52] Wei M.Y., Jacobson T.A. (2011). Effects of eicosapentaenoic acid versus docosahexaenoic acid on serum lipids: A systematic review and meta-analysis. Curr. Atheroscler. Rep..

[53] Jacobson T.A., Glickstein S.B., Rowe J.D., Soni P.N. (2012). Effects of eicosapentaenoic acid and docosahexaenoic acid on low-density lipoprotein cholesterol and other lipids: A review. J. Clin. Lipidol..

[54] Mori T.A., Burke V., Puddey I.B., Watts G.F., O’Neal D.N., Best J.D., Beilin L.J. (2000). Purified eicosapentaenoic and docosahexaenoic acids have differential effects on serum lipids and lipoproteins, LDL particle size, glucose, and insulin in mildly hyperlipidemic men. Am. J. Clin. Nutr..

[55] Abbott K.A., Burrows T.L., Thota R.N., Acharya S., Garg M.L. (2016). Do omega-3 PUFAs affect insulin resistance in a sex-specific manner? A systematic review and meta-analysis of randomized controlled trials. Am. J. Clin. Nutr..

[56] Endres S., Ghorbani R., Kelley V.E., Georgilis K., Lonnemann G., van der Meer J.W., Cannon J.G., Rogers T.S., Klempner M.S., Weber P.C. (1989). The effect of dietary supplementation with n-3 polyunsaturated fatty acids on the synthesis of interleukin-1 and tumor necrosis factor by mononuclear cells. N. Engl. J. Med..

[57] Akchurin O.M., Kaskel F. (2015). Update on inflammation in chronic kidney disease. Blood Purif..

[58] Yoshino J., Smith G.I., Kelly S.C., Julliand S., Reeds D.N., Mittendorfer B. (2016). Effect of dietary n-3 PUFA supplementation on the muscle transcriptome in older adults. Physiol. Rep..

[59] Jankowska M., Cobo G., Lindholm B., Stenvinkel P. (2017). Inflammation and Protein-Energy Wasting in the Uremic Milieu. Contrib. Nephrol..

[60] Rice H.B., Bernasconi A., Maki K.C., Harris W.S., von Schacky C., Calder P.C. (2016). Conducting omega-3 clinical trials with cardiovascular outcomes: Proceedings of a workshop held at ISSFAL 2014. Prostaglandins Leukot. Essent. Fatty Acids.

[61] Jeromson S., Gallagher I.J., Galloway S.D.R., Hamilton D.L. (2015). Omega-3 Fatty Acids and Skeletal Muscle Health. Mar. Drugs.

